# Defining novel functions for cerebrospinal fluid in ALS pathophysiology

**DOI:** 10.1186/s40478-020-01018-0

**Published:** 2020-08-20

**Authors:** Koy Chong Ng Kee Kwong, Arpan R. Mehta, Maiken Nedergaard, Siddharthan Chandran

**Affiliations:** 1 UK Dementia Research Institute at University of Edinburgh, Edinburgh, UK; 2 Centre for Clinical Brain Sciences, University of Edinburgh, Edinburgh bioQuarter, Chancellor’s Building, 49 Little France Crescent, Edinburgh, EH16 4SB UK; 3 Euan MacDonald Centre for MND Research, University of Edinburgh, Edinburgh, UK; 4 Anne Rowling Regenerative Neurology Clinic, University of Edinburgh, Edinburgh, UK; 5 Nuffield Department of Clinical Neurosciences, University of Oxford, Oxford, UK; 6 Center for Translational Neuromedicine, Faculty of Health and Medical Sciences, University of Copenhagen, Copenhagen, Denmark; 7 Center for Translational Neuromedicine, University of Rochester Medical Center, Rochester, NY USA; 8 Centre for Brain Development and Repair, inStem, Bangalore, India

**Keywords:** Glymphatic system, Cerebrospinal fluid, Amyotrophic lateral sclerosis, Motor neuron disease, Frontotemporal dementia, Ageing

## Abstract

Despite the considerable progress made towards understanding ALS pathophysiology, several key features of ALS remain unexplained, from its aetiology to its epidemiological aspects. The glymphatic system, which has recently been recognised as a major clearance pathway for the brain, has received considerable attention in several neurological conditions, particularly Alzheimer’s disease. Its significance in ALS has, however, been little addressed. This perspective article therefore aims to assess the possibility of CSF contribution in ALS by considering various lines of evidence, including the abnormal composition of ALS-CSF, its toxicity and the evidence for impaired CSF dynamics in ALS patients. We also describe a potential role for CSF circulation in determining disease spread as well as the importance of CSF dynamics in ALS neurotherapeutics. We propose that a CSF model could potentially offer additional avenues to explore currently unexplained features of ALS, ultimately leading to new treatment options for people with ALS.

## Introduction

Amyotrophic lateral sclerosis (ALS) is a rapidly progressive fatal neurodegenerative disorder characterised by the selective death of motor neurons. Although the underlying cause of ALS is unknown, recent discoveries in the genetics and molecular pathology of ALS have provided important new insights. These include the finding that monogenetic causes of ALS—accounting for approximately 10% of cases—are phenotypically and pathologically largely indistinguishable from sporadic ALS. Furthermore, over 97% of ALS cases and half of frontotemporal dementia (FTD) are pathologically defined by cytoplasmic mis-accumulation of insoluble TDP-43, leading to these disorders being classified as TDP-43 proteinopathies [135].

Although much remains to be established about ALS pathophysiology, multiple mechanisms are implicated including proteostasis, glutamate excitotoxicity, dysregulation of RNA metabolism, nuclear-cytoplasmic transport and autophagy [16, 36, 73]. Notwithstanding these advances in our mechanistic understanding of ALS, a number of key questions remain unanswered. These include the primary cause of the disease and the significance of ageing as a risk factor, as well as a male predilection [8, 107].

The glymphatic system has recently been recognised as an important clearance pathway for the brain, playing a major role in the regulation of brain metabolites, including glucose and lipids [86]. Notably, its involvement in protein homeostasis has led to it receiving considerable attention in neurodegenerative diseases, particularly Alzheimer’s disease. ALS, despite also being a proteinopathy, has received comparatively less attention in the context of glymphatic function.

Against this background we describe the various potential roles of cerebrospinal fluid (CSF) in ALS pathophysiology, including as a possible driver for the disease process. We start by providing a brief overview of CSF circulation, followed by a primer on the glymphatic system, including the different factors affecting its function (Box 1), both of which have been comprehensively described by excellent reviews [21, 86]. We then assess the possibility of CSF contribution in ALS by considering various lines of evidence.

Box 1: Factors affecting glymphatic function **Arterial pulsation**

Current evidence suggests that exchange between the cerebrospinal and interstitial fluid (CSF-ISF) is mainly driven by arterial pulsation. Reducing arterial pulsation through internal carotid artery ligation resulted in significantly reduced exchange between CSF and ISF [79]. Accordingly, enhancing CSF pulsation by systemic administration of dobutamine was also found to increase CSF influx [79]. It was later demonstrated that CSF flow velocity could be reduced by hypertension [122], a finding confirmed by a more recent study showing impaired glymphatic function in spontaneously hypertensive rats [131]. The reduced CSF flow has been attributed to stiffening of the arterial wall, altering the pulsatility of the arterial wall and leading to increased backflow of blood.

**Respiration**

Neuroimaging results have also revealed both rostrally- and caudally-directed CSF flow produced by inspiration and expiration respectively [211], with forced inspiration triggering greater CSF movement compared to the cardiac cycle-associated flow [52]. Additional support for the contribution of respiration stems from more recent findings demonstrating respiration-driven magnetic resonance encephalography (MREG) pulse waves, which likely reflects glymphatic flow in the human brain [93]. Direct evidence for the link between respiration and CSF-ISF exchange has, however, not yet been established. The contribution of natural breathing to CSF dynamics is also unclear.

**Sleep**

Sleep has been shown to play a major role in waste clearance from the brain. Injected radiolabelled amyloid-beta was found to be cleared much more rapidly in sleeping mice than in awake mice [208]. Sleep is associated with reduced norepinephrine levels, which, in turn, possibly trigger an increase in interstitial space volume, thus reducing resistance to CSF inflow into the brain parenchyma [208]. In humans, sleep deprivation leads to an accumulation of amyloid-beta [180], which could possibly underlie the link between sleep and Alzheimer’s Disease [113]. Another factor involved in waste clearance is body posture, with a lateral or supine posture enabling greater CSF-ISF exchange [99].

**Ageing**

Early suggestions for the impact of ageing on CSF dynamics arose when several studies observed changes in resistance to CSF outflow, secretion and turnover in older individuals [7, 148, 154]. Ageing was subsequently shown in mice to result in decreased CSF inflow into the brain parenchyma and therefore reduced CSF-ISF exchange [95]. Although interstitial space volume was not significantly reduced in aged mice, decreased arterial pulsation along with abnormal AQP4 polarisation were observed, accounting for the decreased CSF inflow into the brain parenchyma.

## Overview of CSF circulation

Surrounding most of the brain and spinal cord, CSF is believed to originate primarily from the choroid plexus, which is an extension of the ependymal lining of the brain ventricles. The majority of CSF is secreted into the two lateral ventricles, from where it converges into the third ventricle through the foramen of Monro, and subsequently flows into the fourth ventricle through the aqueduct of Sylvius. CSF then leaves the fourth ventricle and reaches the subarachnoid space via the foramen of Magendie and the two foramina of Luschka, with an indeterminate fraction of CSF also thought to flow into the central canal of the spinal cord.

CSF drainage however remains a topic of debate, with recent studies challenging the traditional view of reabsorption into dural venous sinuses by arachnoid granulations, which are outgrowths of the arachnoid mater [23, 163]. Alternative exit routes have been evidenced and include the olfactory route and meningeal lymphatics [12, 88, 109, 133, 151], although their relative contributions have not yet been established.

The total volume of CSF in an adult is widely estimated to be about 150 mL, with the larger majority of this volume distributed in the subarachnoid spaces and about 25 mL present in the brain ventricles. Renewal of CSF takes place three to four times in a single day. Whilst the primary role of CSF has long been considered to be the provision of buoyant support to the brain and protection against mechanical damage, another important function of CSF, which will be the focus of this review, is the clearance of metabolic waste products.

## A primer on the glymphatic system

**Fig. 1:**
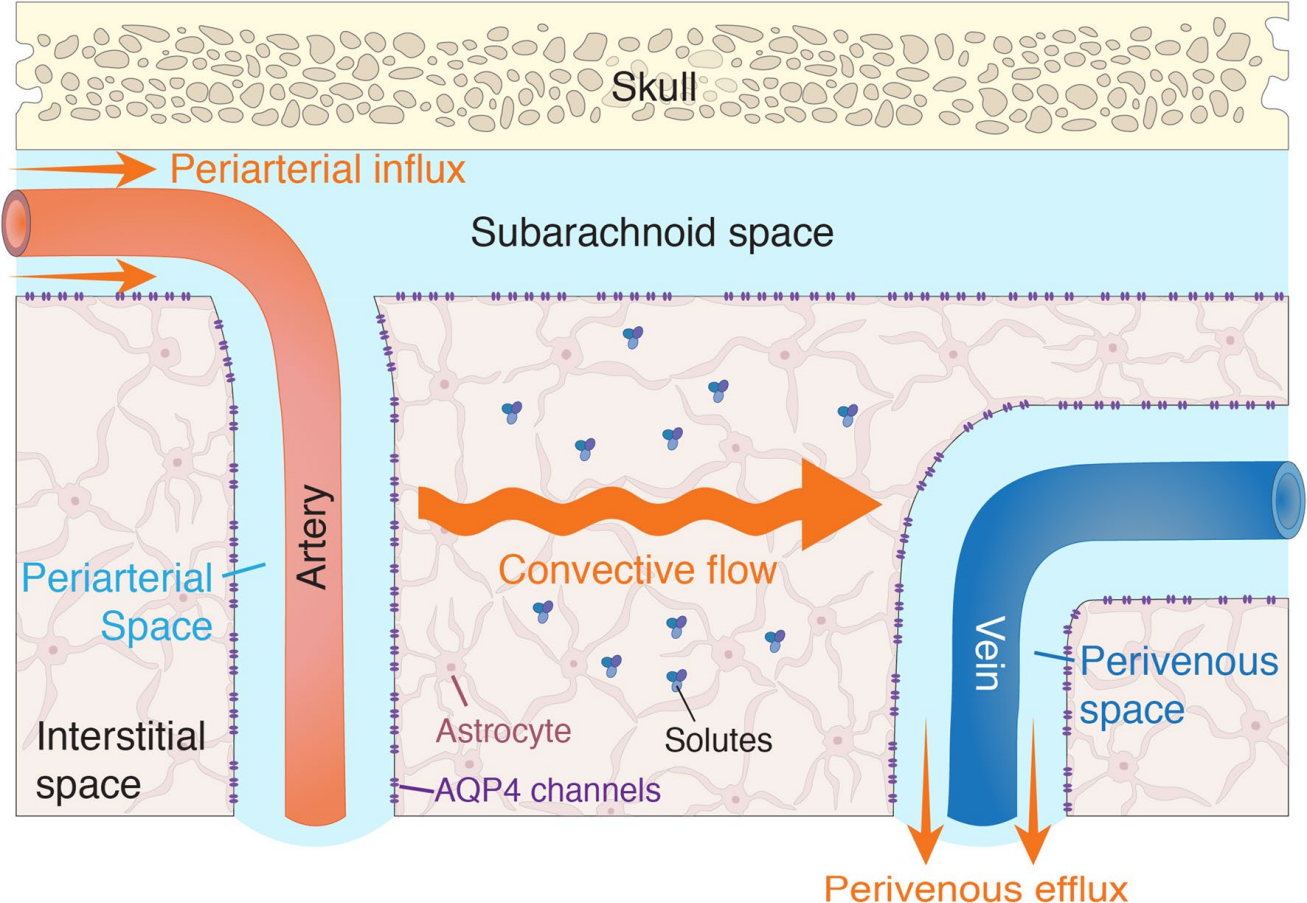
Overview of the glymphatic system. A combination of various forces, including vascular pulsation and respiration, drives the influx of CSF from the subarachnoid space into the periarterial space, or Virchow-Robin space. CSF then moves into the interstitial space, with its entry being promoted by AQP4 channels lining the astrocyte endfeet. A convective flow drives ISF towards the perivenous spaces, carrying solutes, including metabolic waste products, along. As ISF moves from the extracellular space into the perivenous space, it can be drained from the CSF circulation through pathways such as the olfactory route. This highly organised system, enabling rapid CSF-ISF exchange, is known as the glymphatic system

The earliest descriptions of perivascular spaces, or Virchow-Robin spaces, arose as early as the mid-1800s, when investigators, including Rudolf Virchow and Charles Robin, observed spaces surrounding blood vessels penetrating the brain parenchyma [202]. However, it was only in the next century, based on findings derived from dye injection experiments, that perivascular spaces first came to be functionally associated with fluid flow [202]. Today, perivascular spaces are widely regarded as an important site of exchange between CSF and interstitial fluid (ISF), underlying what is now known as the glymphatic system (Fig. 1).

Anatomically, these perivascular spaces are formed by pial arteries that perforate the brain parenchyma after traversing the subarachnoid space. As these pial arteries transition into penetrating arterioles, CSF from the subarachnoid space also extends into the brain parenchyma, bordering the blood vessels and creating CSF-filled spaces that are able to interact with the extracellular space [86, 217]. These perivascular spaces are themselves surrounded by a leptomeningeal layer, which, on one side, adheres to the blood vessel wall, and, on the other side, extends into the pia mater. Importantly, the outer wall of the perivascular space facing the brain parenchyma is lined by astrocyte endfeet expressing AQP4 channels [86]. It should, however, be noted at this point that the precise anatomy of perivascular spaces is still controversial, and much remains to be established about certain key aspects, including the exact fluid flow pathways and the interconnections between different compartments.

**Fig. 2:**
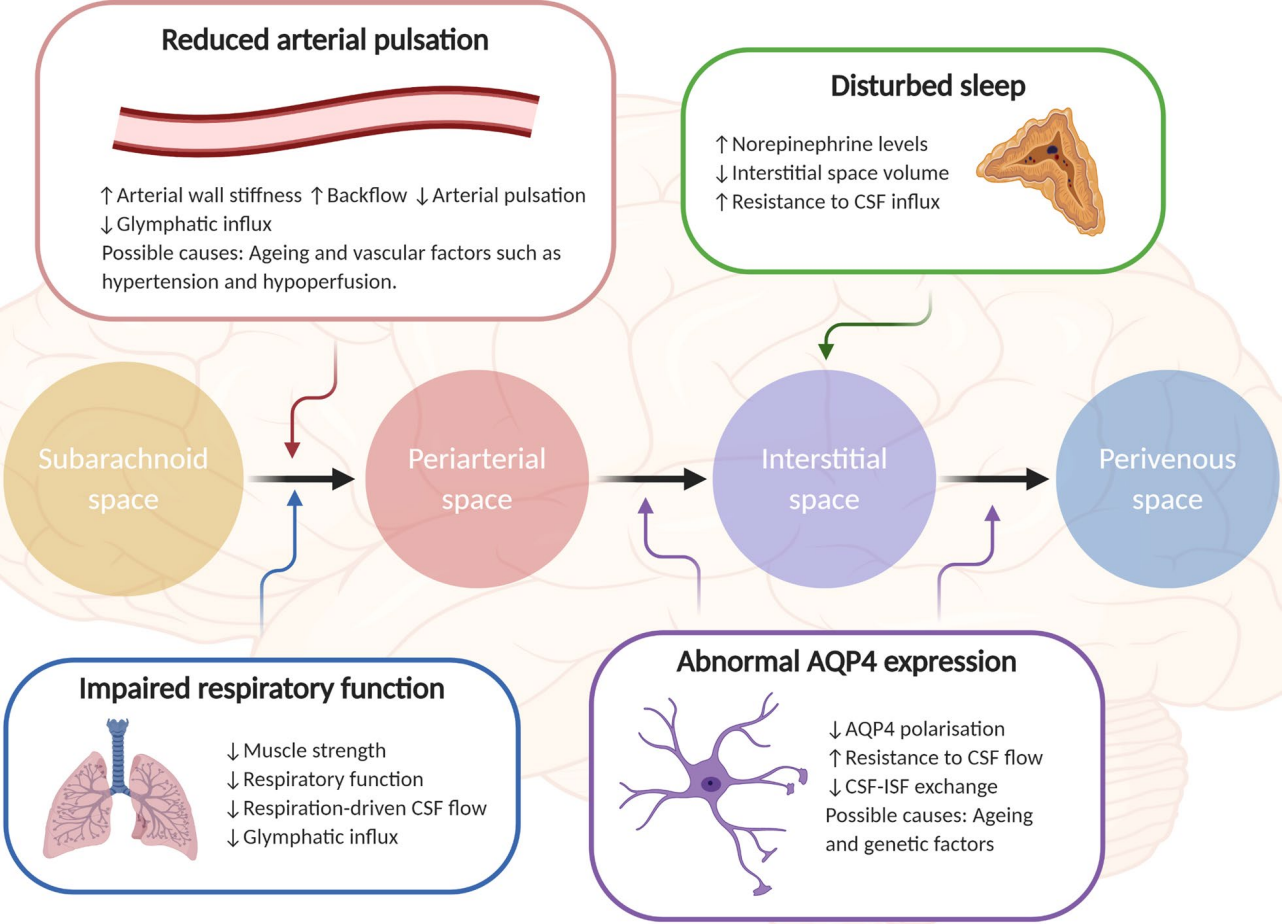
Factors affecting CSF dynamics in ALS patients. Several components of the glymphatic pathway are potentially disrupted in ALS patients. Ageing, for instance, is linked to reduced vascular pulsation, due to an increase in vessel wall stiffness. ALS patients also tend to suffer from poor sleep and impaired respiratory function, particularly towards later stages of the disease. Impairment of two major drivers of glymphatic influx, coupled to disturbed sleep, could underlie highly reduced glymphatic clearance in ALS patients. Other features of ALS that could impact on glymphatic function include abnormal AQP4 expression, raised norepinephrine levels, and vascular factors, such as hypertension and hypoperfusion. Whilst neuroimaging studies have demonstrated abnormal CSF dynamics in ALS patients, further studies may be required to specifically determine the influence of these different factors on glymphatic clearance in ALS patients

Whilst evidence for the role of perivascular spaces in solute transport from the brain interstitium had already emerged in the 1980s [162], the importance of the glymphatic system was only recognised relatively recently, following a landmark study by Iliff et al. who injected fluorescent tracers into mice and characterised the flow of CSF using two-photon microscopy [80]. CSF from the subarachnoid space is first forced into the perivascular space, driven by processes including vascular pulsation, respiration and sleep (Fig. 2). Following this, AQP4 channels lining the astrocytic endfeet are believed to promote the influx of CSF into the brain parenchyma by reducing resistance to inflow [80]. Although the role of AQP4 channels in CSF-ISF exchange has been challenged [181], recent findings from *AQP4*-knockout mice support the importance of AQP4 channels in glymphatic transport and amyloid-beta clearance [121]. Following CSF influx into the extracellular space, a convective flow transports interstitial solutes from the periarterial to the perivenous end. Once ISF reaches the perivenous space, it can subsequently be drained through one of the previously described outflow paths. This elaborate clearance system, also shown to be impacted by ageing (Box 1), has thus been termed the ‘glymphatic system’, given its similarity with the lymphatic system and its reliance on glial cells, in this case, astrocytes.

Despite past studies usually focussing on either the glymphatic system or the meningeal lymphatic system, increasing evidence supports the view that these two systems could be interdependent, with recent findings demonstrating a decrease in glymphatic influx and efflux of interstitial solutes following disruption of the meningeal lymphatic vasculature [47]. Similar to the glymphatic system, meningeal lymphatic function was found to be reduced in ageing, whilst also being linked to neurological disorders, such as Alzheimer’s disease and Parkinson’s disease [5, 47, 112, 220]. We refer readers to previous review articles for a more in-depth discussion of the meningeal lymphatic system [46, 108].

## CSF toxicity in ALS

Although CSF from ALS patients is now known to be constitutionally abnormal [18, 27], with raised levels of proteins, including TDP-43 and neurofilaments [115, 210], its toxicity started being recognised early on, when it was shown to significantly reduce the survival of rat primary neuronal cultures [45]. Since then, numerous studies have been performed on various cell types, including NSC-34 cell lines, and, more recently, hESC-derived and iPSC-derived motor neurons, showing greater degeneration when the cells were exposed to CSF from ALS patients than to CSF from control patients [33, 136, 189, 197]. Interestingly, ALS-CSF was also shown in NSC-34 cells to result in TDP-43 mislocalisation to the cytoplasm, a feature that could be reversed by VEGF [174]. Nevertheless, the exact mechanism by which ALS-CSF induces neuronal degeneration remains to be established, although processes such as excitotoxicity and mitochondrial dysfunction have been suggested [175, 179].

The in vitro effect of ALS-CSF also extends to both astrocytes and microglia, demonstrating a non-cell autonomous component to CSF toxicity. Upon exposure to ALS-CSF, astrocytes undergo a change in morphology, accompanied by increased GFAP reactivity, further acquiring a neuroinflammatory profile [126]. Release of inflammatory markers is also common to microglia exposed to ALS-CSF [127]. Furthermore, rat primary motor neurons co-cultured with glia responded differently to ALS-CSF than when cultured alone [17], adding to the growing body of literature evidencing the role of glia in mediating neurotoxicity [214].

Supporting these in vitro findings, several studies have also reported various changes caused by ALS-CSF in animal models. Neurofilament phosphorylation was observed in rat motor neurons following injection of ALS-CSF into the spinal subarachnoid space [160]. Moreover, intrathecal injection of ALS-CSF led to changes in the Golgi complex [158], potentially affecting protein trafficking and causing endoplasmic reticular stress [196]. Histologically, close similarities were observed between tissue exposed to ALS-CSF and sporadic ALS cases [68]. ALS-CSF was also found to produce phenotypic changes, with rats subjected to intraventricular injections experiencing motor dysfunction [165]. Muscular atrophy was observed in a different study, possibly arising through motor neuron degeneration [173].

Accepting inherent limitations in patient studies, including sample heterogeneity, and that CSF concentration may not reflect cellular levels, these in vitro and in vivo studies nonetheless show that ALS-CSF is toxic to neurons and possesses pro-inflammatory properties [136]. The in vivo finding of toxicity distant to the site of CSF injection also raises a potential role for CSF in disease spread [68], a possibility supported by recent findings demonstrating the onset of motor and cognitive decline, as well as TDP-43 proteinopathy, following ALS-CSF infusion in mice [125].

## Altered CSF dynamics in ALS patients

Evidence for the disruption of CSF flow in ALS includes findings from a phase-contrast electrocardiography-triggered MRI study, which revealed different CSF dynamics in ALS patients, with a delay in CSF flow upon systole and a higher maximum velocity [169]. Abnormal CSF dynamics in ALS patients is also supported by a more recent study demonstrating reduced CSF flow magnitude along with a greater pulse wave velocity, although the causes, as well as implications, of these findings remain to be established [168].

**Fig. 3:**
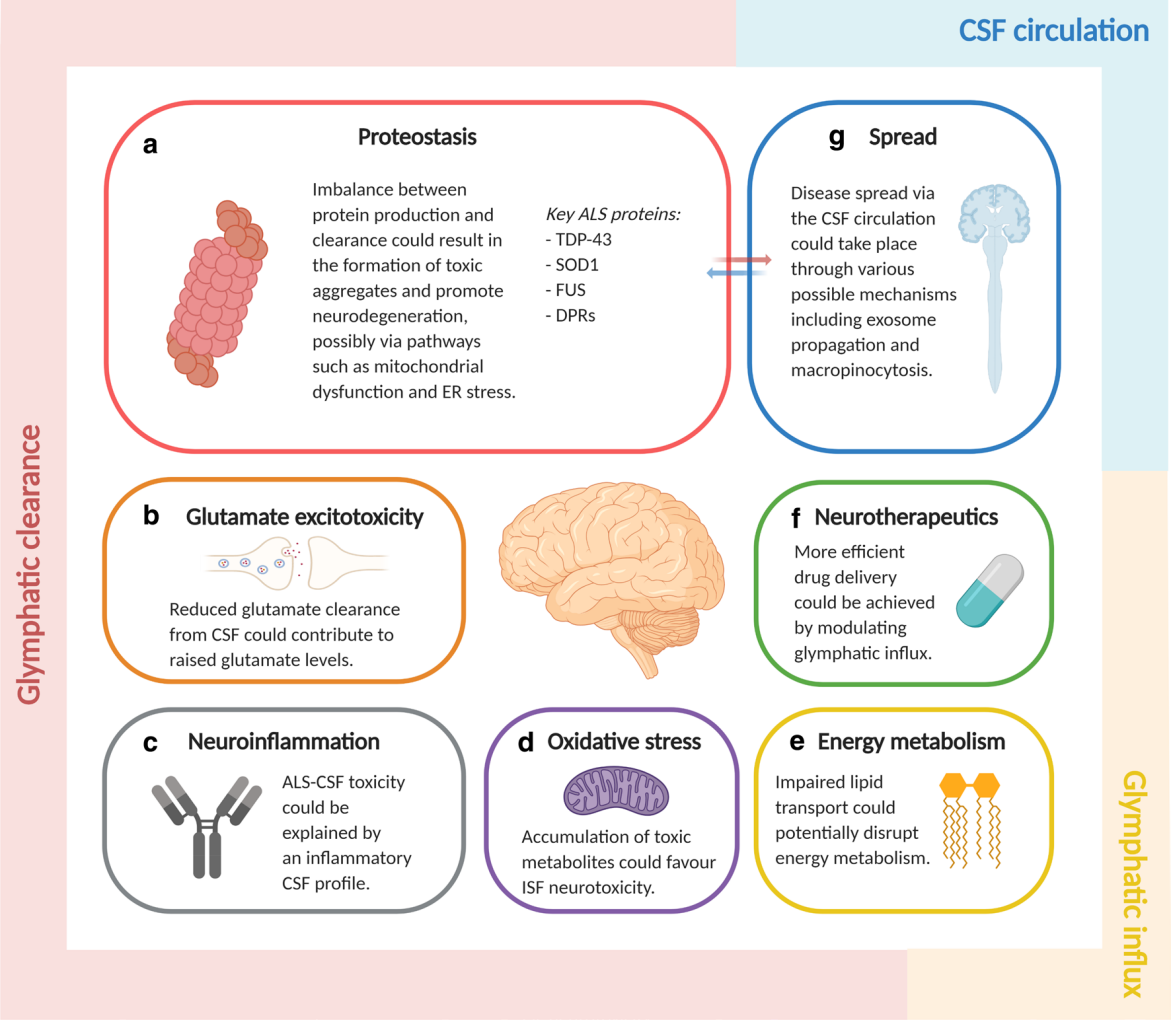
Possible roles for cerebrospinal fluid in ALS. Abnormal CSF dynamics could have important implications in ALS, with both glymphatic clearance and glymphatic influx possibly involved. **a**–**d** Reduced glymphatic clearance could result in the accumulation of various neurotoxic factors, notably that of major pathogenic proteins, including TDP-43 and SOD1. A rise in the levels of CSF components such as glutamate, inflammatory factors and other toxic metabolites could also favour an increasingly toxic interstitial environment (ISF: interstitial fluid). **e** Given the importance of glymphatic function in lipid transport, impaired clearance or influx may possibly affect the regulation of lipid metabolism. **f** Impaired glymphatic influx could also influence pharmacokinetics, particularly in the context of intrathecally administered drugs, and may therefore deserve investigation in ALS neurotherapeutics. **g** Lastly, various lines of evidence suggest that the CSF circulation could act as an important medium for the spread of the disease, with a possible link to proteostasis. *Note* Shape sizes are weighted by their relative significance

Additionally, with human neuroimaging studies revealing changes in CSF dynamics with age [166, 170] and recent evidence also suggesting reduced glymphatic clearance in older individuals [216], a major proportion of ALS patients, given the late onset of ALS, likely experience altered CSF flow and impaired glymphatic function. Sleep, which plays an important role in glymphatic clearance, is also known to be disrupted in ALS patients, who often have apnoea, or sleep poorly, owing to muscle cramps and fasciculations [28]. Although sleep has yet to be acknowledged as a risk factor for ALS, it remains strongly associated with disease severity [4, 28, 157]. Poor sleep, coupled to the severely affected respiratory function in ALS patients, could therefore significantly impair CSF dynamics and therefore glymphatic clearance.

The finding of increased norepinephrine concentration in CSF from ALS patients is also intriguing [22, 35]. Norepinephrine reduces the extracellular space volume and thus increases resistance to glymphatic influx into the brain parenchyma [208]. Of further interest is that whilst sleep quality and respiratory function are known to be negatively correlated with ALS severity [4], a higher norepinephrine level has also been linked to more severe symptoms in FTD [58].

## Abnormal vascular changes in ALS

Vascular function, which is intimately connected to the glymphatic system, is known to be affected in ALS. Multiple studies employing single-photon emission computed tomography (SPECT) have demonstrated hypoperfusion in the brain of both ALS and FTD patients, with greater involvement of the frontal and temporal lobes [1, 82, 199]. Abnormal vascular changes in ALS and FTD have further been evidenced through arterial-spin coupling [53, 177]. In the SOD1 mouse model, blood-spinal cord barrier (BSCB) breakdown may precede hypoperfusion [215], a feature which has previously been linked to disease severity in ALS patients [2, 40].

Genetic association studies linking ALS risk with angiogenic genes, *ANG *and* VEGF*, provide further indirect evidence of the role of vascular factors in ALS [61, 66, 70, 147, 195]. *VEGF* has been heavily implicated in ALS pathophysiology, with several studies demonstrating the protective effect of VEGF therapy on neuronal death, both in vitro and in vivo [15, 98, 187, 198]. Lack of the *VEGF* hypoxia-response element in mice also results in motor neuron degeneration [145]. *VEGF*-associated neurodegeneration has been suggested to arise from ischaemia following reduced vascular perfusion, although this has yet to be established.

The link between hypertension and ALS is currently less clear. Multiple associations have been made between hypertension and ALS onset and survival [116, 129], but contradicting results have also been obtained by other studies [76, 94, 128]. This could possibly be explained by variations in methodology, and further studies are therefore required to confirm this association. Although one study found the use of angiotensin-converting enzyme inhibitors to be linked to a reduced risk of ALS [105], this was not replicated by a later study [63]. Nevertheless, the different vascular aspects of ALS could constitute a further impediment to proper CSF dynamics in ALS patients.

## AQP4 channels and ALS

Overexpression of AQP4 in ALS is well documented in rodent models [19, 48, 137]. In addition to increased expression, reduced AQP4 polarisation with decreased endfeet localisation has also been observed in the SOD1 mouse model [48]. The exact implications of these changes in neuronal degeneration are currently unclear. However, given the various roles played by AQP4 channels, several downstream consequences have been suggested, including loss of blood-brain barrier (BBB) integrity, glutamate dysregulation and impaired potassium homeostasis [219]. The disrupted AQP4 polarisation could also contribute to impaired glymphatic function, commensurate with the features observed in aged mice [95].

Another intriguing finding is that reduced AQP4 expression in *SOD1 *mice triggered earlier disease onset and reduced survival [204]. One explanation suggested by the authors was that the absence of AQP4 channels led to reduced SOD1 clearance, favouring an increasingly neurotoxic extracellular environment. Notwithstanding this, further studies are required to confirm whether such changes in AQP4 expression and their possible implications in glymphatic clearance are features specific to *SOD1* ALS.

## The implications of abnormal CSF dynamics in ALS

Taken together, there are multiple and converging lines of evidence that suggest that the glymphatic system plays a role in ALS pathophysiology. We therefore outline several key processes in ALS that might be impacted by a dysregulated glymphatic system (Fig. 3).

## Proteostasis

Accumulation of various proteins that may directly or indirectly be toxic in ALS is well-established (Box 2). These include mis-accumulated phosphorylated TDP-43, SOD1 aggregates and dipeptide repeats (DPRs) found in the most common inherited form of ALS, due to a repeat expansion mutation in *C9ORF72.* TDP-43 proteinopathy is known to be the most prevalent and has been implicated in many pathways, including mitochondrial dysfunction, autophagy dysregulation and impaired endocytosis [153].

The reasons for accumulation could reflect excess production and/or impaired clearance through autophagy and the ubiquitin-proteasome system (UPS). These are both involved in TDP-43 and SOD1 clearance [89, 101, 212], with another mechanism promoting TDP-43 clearance being the more recently discovered endolysosomal pathway [101, 106]. Thus, indirect evidence from rare inherited forms of ALS due to mutations in genes linked to autophagy or the UPS, such as *VCP, OPTN* and *UBQLN2,* argues for an important role for dysregulated clearance in ALS. Clear evidence supporting the overproduction of TDP-43 in ALS, however, remains to be established.

Given the established role of the glymphatic system in protein clearance, impairment of glymphatic function could also contribute to the accumulation of the different toxic proteins in ALS. The presence of TDP-43, SOD1 and DPRs in ALS-CSF is well-evidenced and points to a potential role for CSF in their regulation. It is therefore possible that ageing, coupled to the different possible sources of glymphatic disturbance already discussed, could result in an imbalance between production and clearance of these pathogenic proteins. Notably, ageing has further been argued to promote the deterioration of processes such as autophagy and the UPS [138], which could further exacerbate protein clearance in the diseased state. The numerous clearance mechanisms involved in amyloid-beta clearance in Alzheimer’s disease [190] also suggest that other major clearance pathways in ALS beyond intracellular mechanisms are yet to be defined, of which the glymphatic system would be one notable example.

Notwithstanding this, protein inclusions associated with ALS, including those enriched in TDP-43, SOD1 and DPRs, are predominantly intracellular, as opposed to amyloid-beta aggregates, found in Alzheimer’s disease, which also reside extracellularly. Thus, glymphatic clearance in the context of ALS could potentially follow from processes such as exosome secretion [34, 78] or necroptosis, in which intracellular contents are released from the dying cell into the interstitial space [83]. A possible interplay between intracellular mechanisms and extracellular processes could also be involved, with UPS clearance, for instance, being influenced by an altered extracellular environment, owing to impaired glymphatic function.

Whilst considerable progress has been made towards understanding ALS pathophysiology, its primary cause has remained a matter of debate, with genetic mutations accounting for only a minority of ALS cases. Hence, the significance of ageing in the impairment of clearance mechanisms could provide a promising avenue to explore in ALS.

Box 2: Key ALS proteins **TAR DNA-binding protein 43 (TDP-43)**

TDP-43 inclusions are now widely considered as the pathological hallmark of ALS, with more than 95% of ALS patients known to exhibit ubiquinated TDP-43 deposits [135]. The TDP-43 protein, which is encoded by the *TARDBP* gene, possesses a wide range of functions, most notably in RNA regulation. About 5% of familial ALS cases can be attributed to mutations in the *TARDBP* gene [221]. TDP-43 aggregation is thought to be implicated in the disease process, and some of the proposed mechanisms for TDP-43 induced cytotoxicity include mitochondrial dysfunction, autophagy dysregulation and impaired endocytosis [153].

**Superoxide dismutase 1 (SOD1)**

*SOD1* was the first ALS-linked gene to be discovered and mutations in the *SOD1* gene are now known to account for about 15% of familial ALS cases and 1% of sporadic cases [221]. The *SOD1* gene codes for the Cu-Zn superoxide dismutase enzyme whose cellular function is to convert superoxide radicals into hydrogen peroxide and oxygen. Although increasing evidence indicates a gain-of-function mechanism for SOD1 pathogenicity [152], its neurotoxic effects are still incompletely understood, with possible explanations including oxidative stress and proteostasis [73]. Considerable evidence also supports the non-cell autonomous nature of SOD1 cytotoxicity, with astrocytes and microglia considered to play an important role [149].

**Fused-in-sarcoma (FUS)**

Another ALS protein known to form cytoplasmic inclusions is the FUS protein. Mutations in the *FUS* gene are responsible for a relatively small fraction of familial ALS cases (about 3%), and an even smaller proportion of sporadic ALS cases [221]. The FUS protein is mostly found in the nucleus and is involved at multiple steps of the RNA processing machinery, such as transcription and splicing [139]. The exact mechanism by which *FUS* mutations promote neurodegeneration is, however, unclear.

**C9ORF72-associated dipeptide repeats (DPRs)**

The commonest known cause of ALS is a repeat expansion in the *C9ORF72* locus, accounting for about 40% of familial ALS and about 10% of sporadic ALS [37, 114]. One feature of *C9ORF72*-mediated ALS is the non-canonical translation of repeated RNA sequences, giving rise to five classes of DPR proteins, namely, poly-GA, poly-GP, poly-GR, poly-PA and poly-PR [16]. Although evidence for their contribution to the disease process is currently mixed, various mechanisms, including caspase activation and inhibition of membrane-less organelles formation, have been implicated [100, 213].

## Glutamate excitotoxicity

Glutamate excitotoxicity has long been viewed as an important pathophysiological mechanism in ALS and FTD. Riluzole, which inhibits glutamate release, is currently the only globally approved drug for ALS [84]. One reason for the particular susceptibility of motor neurons to AMPA-mediated glutamate excitotoxicity is thought to be their low buffering ability upon calcium influx [92]. This influx is promoted by permeability of the AMPA receptor, which can be affected by changes to its subunit composition. For instance, reduced expression of the impermeable GluR2 subunit results in an increased influx of calcium ions, potentially contributing to neurodegeneration in ALS [91, 194].

Glutamate excitotoxicity could also be caused by direct overstimulation of glutamate receptors, due to an increased level of extracellular glutamate [92]. Although increased synaptic release has been suggested to contribute to raised glutamate levels, strong evidence supports the involvement of the glutamate transporter EAAT2, with studies demonstrating reduced EAAT2 expression in different ALS models, including in *SOD1* mice [64, 77, 164, 167, 203]. Intriguingly, a minor increase in glutamate levels has also been found to trigger neurodegeneration [29, 71, 103], even when raised to levels comparable to that of ALS-CSF [42].

Whilst the cause of increased glutamate levels could result from several processes, the role of CSF in glutamate regulation [6] could indicate a possible contribution in promoting potentially neurotoxic levels of glutamate in ALS [62, 185]. This could again be driven by the different aspects of ALS that affect CSF dynamics, including ageing, sleep disruption and vascular factors, amongst others. It should, however, be noted that CSF glutamate levels may not necessarily reflect extracellular levels, although increased CSF glutamate concentrations could likely affect exposed astrocytes.

## Energy metabolism

Dysregulated energy metabolism is now a widely known feature of ALS, with patients generally suffering from weight loss, hypermetabolism and hyperlipidaemia [55]. Although the energy imbalance in ALS could stem from several processes, two important contributing factors are thought to be the higher basal energy expenditure in ALS patients [50, 65], and reduced nutrition due to dysphagia [96]. The causes of hyperlipidaemia, which could further affect energy metabolism, also remain controversial [55]. Along with increased apoE levels [97] and type 2 diabetes [85, 118], hyperlipidaemia has in fact been shown to be protective in ALS [51, 54]. Nevertheless, the link between energy metabolism and ALS pathogenesis remains unclear despite animal studies providing a number of suggestions [55] with therapeutic promise [119].

Hence, given the recently characterised role of the glymphatic system in lipid transport [159], an impairment in CSF circulation could potentially be a further underlying cause of the abnormal energy metabolism observed in ALS. BBB impermeability largely restricts influx of lipids and cholesterol, which are believed to be produced within the CNS by the choroid plexus and astrocytes [3, 31, 59, 209], with excess cholesterol being released into the blood circulation [24, 111]. Following synthesis, apoE is observed to distribute through the perivascular space in order to reach the brain [159]. We speculate that impaired lipid regulation by the glymphatic system, coupled to increased BBB permeability in ALS, may well disrupt energy metabolism. This is supported by numerous studies demonstrating an altered CSF metabolome in ALS patients [25, 26, 69, 207]. Again however, whether these changes could contribute to ALS pathogenesis is difficult to answer.

## Neuroinflammation

To date, several studies have helped to demonstrate the inflammatory profile of ALS-CSF, revealing raised levels of various immune components, including C3c, albumin and IgG in ALS-CSF [10, 11, 44, 67, 102]. Out of these, IgG, also present in post-mortem ALS samples, has been found to favour neurodegeneration, both in vitro and in vivo [49, 155, 156]. IgG further possesses pro-inflammatory properties, promoting microglia recruitment and upregulation of inflammatory cytokines [124, 141, 142]. Some of the other chemokines found to be elevated in ALS-CSF include IL-6, TNF-α and TGF-β [81, 130, 172], with the finding of raised TNF-α levels being particularly interesting, given its associations with glutamate excitotoxicity [43, 178, 218]. Whilst their direct involvement in ALS pathophysiology is still unknown, the presence of these different immune factors in ALS-CSF, some of which have been shown to be neurotoxic, could suggest an important role for CSF in neuroinflammation, with the neurotoxic environment possibly favoured by altered CSF dynamics and reduced clearance. This would in fact provide a potential explanation for the well-evidenced toxicity of ALS-CSF, which has, in turn, been suggested to possibly contribute to ALS pathophysiology [176]. Further studies are nevertheless required to confirm this association.

## Oxidative stress

Given their high metabolic rate as well as their inability to divide, motor neurons could be particularly vulnerable to accumulation of toxic metabolites, including lactate, 4-hydroxynonenal (HNE) and peroxynitrite [176]. High lactic acid levels have been shown to induce neuronal degeneration and promote inflammation [72, 134], whilst HNE, a marker of lipid peroxidation, results in oxidative stress in cultured motor neuron hybrid cells at levels similar to those of ALS-CSF [183]. Peroxynitrite, although controversial, has also been suggested to promote oxidative stress [191]. It is therefore possible that CSF, which is known to regulate the level of metabolites, including that of lactate [75, 110], could potentially help to prevent the accumulation of waste products that would otherwise result in a highly neurotoxic extracellular environment.

## CSF circulation and spread

The variable sites of disease onset and subsequent disease evolution have raised the question as to what determines ALS disease spread. This is unknown and debate has hitherto focussed on questions of anterograde or retrograde spread [41, 57]. Some general patterns can, however, be noted. Specifically, the disease is localised focally during early stages and spreads outwards in a contiguous pattern. The spread is more often directed caudally than rostrally. Additionally, the pattern of spread is heterogeneous in nature, making it difficult to predict [161].

More recently, a growing body of literature suggests that ALS spread could be explained by a prion-like mechanism. Notably, the mutant SOD1 protein, which has been demonstrated to possess high fibril-forming propensity under certain conditions, can promote fibrillation in wild-type SOD1 [39]. TDP-43 is also able to form aggregates in vitro, although the factors that predispose TDP-43 to aggregation are still incompletely understood [153]. Evidence further indicates that these properties could be shared by FUS [140] as well as DPRs [205]. These observations suggest a close similarity with other neurodegenerative diseases, such as Alzheimer’s disease and Parkinson’s disease, which have also been characterised as proteinopathies.

Although the prion-like properties of the different ALS proteins are starting to gain acceptance within the scientific community, less is known about how intercellular spread could take place in vivo. Trans-synaptic transmission has been suggested as one possible mechanism [13, 32]. Another possibility is through exosome propagation. It has in fact been shown that SOD1-containing exosomes could be secreted into the extracellular space [192], with possible uptake by macropinocytosis [132]. There is evidence that TDP-43 could also spread via exosomes [60], although further studies are required to establish this. Another recently proposed model is that of corticofugal axonal spread, for which strong support arises from the particular vulnerability of neurons receiving connections from pyramidal cells of the neocortex [32].

A further pathway already described by others is that spread of the disease could occur via the CSF circulation [182]. The presence of misfolded proteins, including TDP-43 and SOD1, has already been demonstrated in ALS-CSF, and, whilst neurons could potentially directly ingest these proteins through phagocytic mechanisms [30], another plausible mechanism previously suggested would be via uptake of exosomes containing the misfolded proteins [90]. Indeed, CSF exosomes from Alzheimer’s disease and Parkinson’s disease patients have been shown to contain alpha-synuclein and misfolded tau respectively [188, 201]. Protein-containing exosomes are also known to be present in ALS-CSF [74] but whether they specifically contain TDP-43 or SOD1 remains to be established.

Hence, whilst we do not propose that spread of the disease occurs solely through CSF pathways, such a mechanism of spread could clearly complement other proposed models. It would also help to explain the non-contiguous pattern of spread and the multifocal pattern of initiation observed in some ALS cases [171], and even provide a potential link between ALS and FTD, by providing a direct route of spread from the brain to the spinal cord. Other arguments have also been made in support of spread via CSF; for instance, the proximity of vulnerable neurons to CSF and also the susceptibility of particular cranial nerves to the disease [182].

## Towards a glymphatic system model in ALS

One major caveat in considering glymphatic involvement in ALS is that its function has so far only been demonstrated in the brain, with its contribution in the spinal cord still unclear. Indeed, spinal cord and brain anatomy differ considerably both in terms of parenchymal and leptomeningeal organisation, suggesting possible mechanistic differences in waste clearance. Whether clearance from the brain and clearance from the spinal cord are influenced by similar factors and could therefore be modulated via similar mechanisms are also not known. These distinctions may therefore need to be further explored, given their possible importance with regard to spinal cord involvement in ALS. Another point to consider is that most studies on the glymphatic system have been carried out in rodents, with definitive evidence for its existence in humans yet to be established. However, results have indicated a high possibility of this being the case, with some studies reporting a number of inter-species similarities, not only in terms of the associated anatomical structures, but also the factors affecting clearance, such as sleep [21, 56, 120, 163, 216].

Hence, the glymphatic system could play an important role in reconciling disease mechanism with epidemiology and other currently unexplained aspects of ALS. It would firstly support the existence of a threshold effect suggested by the significance of ageing as a risk factor. Secondly, it could help to explain the intriguing gender disparity in ALS, with the established protective influence of oestrogen in preserving vascular, and therefore glymphatic, function possibly accounting for the lower female incidence. This could further underlie the postmenopausal increase in female:male ratio observed by some studies [38, 117]. Adopting a glymphatic system model could not only suggest a primary, or at least contributory, cause for the disease, but also offer potential explanations for the clinical features of ALS, including its progression and heterogeneity.

Whether the intriguing difficulty in generating *C9ORF72* mouse models that recapitulate the disease phenotype bears a connection to glymphatic function also deserves mention. Both CSF production and turnover rate are much greater in mice than in humans [87], and when coupled to the significantly higher basal heart rate in mice, have been suggested to result in improved waste clearance and therefore a reduction in the accumulation of toxic products [21]. This could possibly explain why *C9ORF72* mouse models have failed to exhibit motor dysfunction. Interestingly, however, cognitive defects have been observed both in mutant TDP-43 and *C9ORF72* mouse models [9, 20], although whether this can be attributed to the comparatively higher metabolic rate inherent to the mouse brain is unclear [21].

Understandably, many areas need to be addressed before acknowledging glymphatic dysfunction as a contributor to ALS pathophysiology. Despite the involvement of certain angiogenic genes, evidence for a clear genetic link between ALS and genes implicated in glymphatic function is still missing. Furthermore, definitive evidence for the significance of sleep [184] and hypertension as risk factors for ALS is also yet to be established. It is also unclear, according to this model, why ALS incidence would decrease above the age of about 70 [8, 107].

## Glymphatic system and ALS neurotherapeutics

Whilst the glymphatic system has more often than not been associated with waste clearance from the brain, regulating glymphatic influx could also significantly impact ALS neurotherapeutics. With BBB impermeability acting as a major hurdle to drug delivery in ALS and other neurological disorders, intrathecal therapy has emerged as a promising treatment modality in recent years. Indeed, administering the drug directly into the cerebrospinal circulation has multiple advantages, such as allowing for more accurate monitoring of drug levels, thereby ensuring better pharmacokinetic results [193]. The obvious downside to intrathecal administration, however, is the risk of complications owing to its invasive nature.

Several intrathecal therapies are currently being trialled in ALS, including anti-sense oligonucleotides (ASOs), mesenchymal stem cells (MSCs) and growth factors, such as brain-derived neurotrophic factor (BDNF), ciliary neurotrophic factor (CNTF) and vascular endothelial growth factor (VEGF) [193]. Promisingly, these have shown positive safety profiles so far, although evidence for their efficacy remains to be established [123, 143, 144, 186]. Based on this, further studies may be required to investigate the influence of CSF dynamics on the pharmacokinetics of intrathecal drugs. Key considerations include whether the intrathecally administered drug is appropriately taken up by the spinal cord, and, perhaps more importantly, whether it can penetrate the brain parenchyma. Pre-clinical studies in mice have indicated widespread transduction following intrathecal delivery [14, 146, 200]. This has, however, been more difficult to confirm in humans, with pharmacokinetic analysis being largely restricted to monitoring CSF drug levels [193].

To further emphasise the significance of achieving more efficient drug delivery, different variables, such as diffusion rate and anatomical constraints, have been suggested to limit drug access to deeper parts of the brain and the spinal cord [206]. Additionally, many of the factors impacting on CSF dynamics in ALS patients, including ageing and hypertension, could potentially affect drug distribution following intrathecal injection. However, studies performed in mice have shown that CSF influx into the brain parenchyma could be improved by administering hypertonic saline or mannitol, which both increase plasma osmolality, or ketamine-xylazine, a modulator of slow-wave activity [150, 208]. A more recent study further demonstrated that dexmedetomidine, an α2-adrenergic agonist, which also affects slow-wave oscillations, could enhance brain distribution following intrathecal delivery [104].

## Conclusion

The glymphatic system has promise in potentially unifying many different aspects of ALS, and may therefore be viewed as a novel paradigm when considering ALS pathophysiology. We suggest that further avenues be explored to specifically examine features of disease onset and spread. Several lines of enquiry from pre-clinical to epidemiological studies are thus required to shed light on the extent of CSF contribution in ALS, with the hope of ultimately identifying new treatment options for people with ALS.

## Data Availability

Not applicable.

## Abbreviations

ADAR: Adenosine deaminases acting on RNA
ALS: Amyotrophic lateral sclerosis
ALS-CSF: CSF from ALS patients
AMPA: α-amino-3-hydroxy-5-methyl-4-isoxazolepropionic acid
ANG: Angiogenin
apoE: Apolipoprotein E
AQP4: Aquaporin 4
ASO: Anti-sense oligonucleotide
BBB: Blood-brain barrier
BDNF: Brain-derived neurotrophic factor
BSCB: Blood-spinal cord barrier
C9ORF72: Chromosome 9 open reading frame 72
CNS: Central nervous system
CNTF: Ciliary neurotrophic factor
CSF: Cerebrospinal fluid
DPR: Dipeptide repeat
EAAT2: Excitatory amino acid transporter 2
FTD: Frontotemporal dementia
FUS: Fused-in-sarcoma
hESC: Human embryonic stem cell
HNE: 4‐hydroxynonenal
IgG: Immunoglobin G
IL-6: Interleukin-6
iPSC: Induced pluripotent stem cell
ISF: Interstitial fluid
MSC: Mesenchymal stem cell
MREG: Magnetic resonance encephalography
NSC-34: Mouse spinal cord-neuroblastoma hybrid cell line
OPTN: Optineurin
SOD1: Superoxide dismutase 1
SPECT: Single-photon emission computed tomography
TDP-43: TAR DNA binding protein 43
TGF-β: Transforming growth factor beta
TNF-α: Tumour necrosis factor alpha
UBQLN2: Ubiquilin-2
UPS: Ubiquitin-proteasome system
VCP: Valosin containing protein
VEGF: Vascular endothelial growth factor
